# EX-vivo whole blood stimulation with A2E does not elicit an inflammatory cytokine response in patients with age-related macular degeneration

**DOI:** 10.1038/s41598-021-87337-1

**Published:** 2021-04-15

**Authors:** Jon Ambæk Durhuus, Maarten P. Rozing, Marie Krogh Nielsen, Christopher Rue Molbech, Guido Keijzers, Morten Scheibye-Knudsen, Lene Juel Rasmussen, Rudi G. J. Westendorp, Torben Lykke Sørensen

**Affiliations:** 1 The Research Unit for General Practice and Section of General Practice, Department of Public Health, University of Copenhagen, Copenhagen, Denmark; 2 Department of Cellular and Molecular Medicine, Center for Healthy Aging, Faculty of Health and Medical Sciences, University of Copenhagen, Copenhagen, Denmark; 3 Clinical Eye Research Division, Department of Ophthalmology, Zealand University, Hospital Roskilde, Roskilde, Denmark; 4 Faculty of Health and Medical Sciences, University of Copenhagen, Copenhagen, Denmark; 5 Department of Public Health, Section of Epidemiology, University of Copenhagen, Copenhagen, Denmark; 6 Department of Cellular and Molecular Medicine, University of Copenhagen, Copenhagen, Denmark; 7 The HNPCC Register, Clinical Research Department, Copenhagen University Hospital, Hvidovre, Denmark

**Keywords:** Medical research, Preclinical research

## Abstract

Age-related macular degeneration (AMD) is a highly prevalent degenerative disease and a leading cause of vision loss worldwide. Evidence for an inflammatory component in the development of AMD exists, yet the exact mechanisms remain unclear. Bisretinoid *N*-retinylidene-*N*-retinylethanolamine (A2E) in retinal pigmental epithelial (RPE) cells, and in extracellular deposits constitutes a hallmark of AMD, but its role in the pathology of AMD is elusive. Here, we tested the hypothesis that A2E is responsible for the heightened inflammatory activity in AMD. To this end, we measured ex vivo mRNA expression of the cytokines TNF-α, IL-6, and IL-10 in whole blood samples after stimulation with A2E in a clinical sample of 27 patients with neovascular AMD and 24 patients with geographic atrophy secondary to AMD. Patients’ spouses (n = 30) were included as non-affected controls. After stimulation with A2E, no statistical differences were found in the median expression level of TNF-α, IL-6, IL-10 between the control group, and the neovascular AMD and the geographic atrophy group. Our findings do not support evidence for the hypothesis, that A2E per se contributes to heightened inflammatory activity in AMD.

## Introduction

Age-related macular degeneration (AMD) is a degenerative disease and a leading cause of irreversible vision loss in older people affecting up to 5% of the population above 75 years in the Western world^1,2^. Risk factors include genetics, environmental factors, and health behaviours with ageing as the main risk factor^3,4^. We have previously described a two-level model to accommodate the diverse pathophysiological mechanisms of AMD^5^. The two-level model explains how various risk factors fit into two diverse, but sequentially operating mechanisms; the random accumulation of damage and the subsequent inflammatory host response. The accumulation of molecular damage corresponds to the first step in the development of AMD. Whether this will develop into late AMD or not, depends on the second step—the subsequent inflammatory host response, which is partially under genetic control^6^. Molecular damage can trigger a strong inflammatory response, which can lead to additional damage. Altogether this can ultimately result in a functional decline. Thus, both components of the two-level model are required for AMD to develop. If the inflammatory host response is less pronounced, the development of AMD will be delayed, potentially to the extent that these never become clinically manifest^5,7^.

The eye was long regarded an immune privileged organ, but emerging evidence has shown that the systemic immune system indeed has a crucial role in the pathophysiology of AMD^8–10^. In AMD, peripheral inflammatory/classical monocytes are recruited to the sites of retinal injury where they differentiate into macrophages^11^. Macrophages express Toll Like Receptors (TLRs) and scavenger receptors, which recognize pathogen-associated patterns (PAMPs), and phagocytosed microorganisms, lipids, and dying cells, as well as aberrant or damaged molecules, which are perceived as danger-associated molecular patterns (DAMPs)^12^. Upon activation by DAMPs or PAMPS, monocytes produce effector molecules such as cytokines and ROS, thereby initiating inflammation^13^. An inflammatory environment skews M1 macrophages to a pro-inflammatory state, where cytokines such as IL-6 and TNF-α are secreted^14^. Higher levels of IL-6 and TNF-α could be relevant in the aetiology of AMD, as these cytokines can contribute to pathological changes in retinal pigmental epithelial (RPE)^15,16^.

Activated neutrophils are important for a functional innate immune system^17^ and optimal tissue maintenance involves neutrophils and monocytes, while late tissue healing involves macrophages, growth factors and pro-inflammatory cytokines. The physiological changes of para-inflammation lead to tissue repair, but a macrophage-driven hyperactive response can result in fibrosis and tissue destruction^18,19^. We have previously found higher expression of the neutrophilic activity marker CD66b in patients with neovascular AMD^20^. Patients with AMD in general, seem to have a higher neutrophil-to-lymphocyte ratio (NLR), which was also concluded in a recent meta-analysis. In patients with GA there was an insignificantly increased NLR. However, a larger and significant increase in patients with neovascular AMD was described, which indicates that NLR are likely to either contribute to AMD at a later stage of disease, or to the formation of neovascularization^21^.

AMD is characterized by reduced RPE function and photoreceptor loss in the macula^22^. Accumulation of drusen and lipofuscin in RPE cells constitutes the hallmark of AMD. The main components of lipofuscin are fluorescent bisretinoids, such as *N*-retinylidene-*N*-retinylethanolamine (A2E). The biogenesis of A2E probably occurs as a result of impaired lysosomal and autophagic digestion of cellular components^23,24^. The exact mechanism how A2E contributes to the development of AMD is unknown. It is conceivable that A2E may act as a DAMP and activate the monocyte TLR. In line with this hypothesis is the circumstantial evidence that A2E has pro-inflammatory properties: A2E treatment of RPE cells induces increased expression of a range of pro-inflammatory cytokines^25–28^. We therefore hypothesise that A2E is responsible for the heightened inflammatory activity in AMD in leukocytes. The inflammatory properties of A2E in RPE cells have previously been suggested by others. Anderson et al., have shown that 25 µM A2E induced increased levels of IL-1ß, IL-2, IL-6, and TNF-α^28^. Zhang et al. also treated RPE cells with 25 µM A2E and found increased cytokine production including IL-6 and IL-10^26^. Considering the important role of systemic monocytes in the development AMD, we considered peripheral leukocytes a useful model to mimic the effects of A2E on innate immune cells in the RPE. Stimulation with bacterial lipopolysaccharide (LPS) and the age-related glycosylated end-product (AGE), Nε-carboxymethyllysine (CML), which are known to elicit a respectively vigorous and moderate cytokine production in whole blood assays^29–31^, were used as positive controls. AGEs have been shown to activate NFκB and contribute to a more reactive pro-inflammatory response^32^. To this end, we measured cytokine mRNA expression in whole blood samples after stimulation with A2E. Our approach was based on a ELISA-based whole blood ex vivo LPS stimulation assay. The assay was developed in order to quantify the capacity of the innate immune response by measuring IL-10 and TNF-α after LPS treatment^33^. By using the whole blood ex vivo LPS stimulation assay it was demonstrated that the observed tenfold variation in inter-individual differences in cytokine secretion was predominantly driven by genetics^6^. We performed our stimulation assay in a population of patients with AMD, containing patients diagnosed with neovascular (“wet”) AMD and geographic atrophy (GA), and compared them with a group of healthy, i.e., non-AMD affected, controls.

## Results

### Patient characteristics

All participants were subjected to a structured interview regarding past and present medical history and medication use. Height and weight were used to calculate body mass index (BMI). We assessed physical activity using a single question which is validated in patients with AMD^34^. Tobacco use was registered as current smokers (smoked within the past year), ever smokers (> 100 cigarettes during their life) or never smokers. For current and former smokers, we registered their self-reported tobacco use in pack years (1 unit equivalent to 20 cigarettes per day for 1 year)^35^. Adjusting analyses for smoking status (current, previous, never) did not materially differ between groups (Table S1) and the same trend was seen with association between smoking pack years (Table S2). Alcohol was registered as self-reported units per week (1 unit = 12 g ethanol), which is unit known to layman in Denmark.

### Patients with AMD shows skewed characteristics when compared to a control series

The demographics and clinical characteristics of the patients with neovascular AMD (N = 30) or GA secondary to AMD (N = 30) and a control series (N = 30) are shown in Table 1. The table shows the baseline characteristics for the control group and the two groups diagnosed with AMD. In both the neovascular AMD and the GA group, median age was higher than in the control group (Mann–Whitney U test P = 0.01 and P < 0.001 respectively). Patients with neovascular AMD and GA were more often smokers or had previously smoked in comparison to controls. The median neutrophil count and the NLR were significantly higher in neovascular AMD (P = 0.026 and 0.001 respectively) as well as the GA group (P = 0.08 and 0.001 respectively) as compared to the control group.

**Table 1:** Clinical characteristics of patients with either with neovascular AMD or geographic atrophy secondary to AMD and a control series.

	Controls (N = 30)	Neovascular AMD (N = 30)	GA (N = 30)	P value
**Demographics**				
Age (years)	69.5 (65.0–75.0)	77.0 (72.3–80.0)	80.5 (75.3–88.8)	** < 0.001**
Females, n (%)	19 (56)	20 (62.5)	19 (59)	0.92
**Co-morbidity**				
Hypertension*,* n (%)	5 (14.7)	13 (40.6)	13 (40.6)	**0.032**
Hypercholesterolemia, n (%)	4 (11.8)	4 (12.5)	5 (15.6)	0.89
Diabetes mellitus type II, n (%)	2 (5.9)	5 (15.6)	4 (12.5)	0.44
**Lifestyle factors**				
BMI (kg/m^2^)	26.8 (23.2–28.9)	25.7 (26.7–28.5)	26.1 (24.2–27.7)	0.42
Alcohol consumption, (n)	3 (1–7)	4.5 (1.25–9.25)	2 (1–6)	0.45
**Smoking status, n (%)**				
Smoker	2 (5.9)	9 (28.1)	4 (12.5)	
Previous smoker	7 (20.6)	14 (43.8)	17 (53.1)	**0.001**
Non-smoker	25 (73.5)	9 (28.1)	11 (34.4)	
Pack years	0 (0–4)	15 (0–35.94)	8.75 (0–22)	**0.001**
**Haematology**				
Platelet cell count ,10 9/L	226 (201–250)	233 (187–272)	222.5 (186–262)	0.24
**WBC count, 10 9/L**				
Lymphocytes, 10 9/L	1.65 (1.40–1.90)	1.60 (1.20–2.10)	1.30 (1.10–1.75)	0.27
Monocytes, 10 9/L	0.50 (0.40–0.70)	0.60 (0.50–0.70)	0.65 (0.50–0.80)	0.36
Neutrophils, 10 9/L	3.05 (2.35–3.65)	3.85 (2.98–4.83)	3.90 (2.90–5.50)	**0.011**
Neutrophils-to-lymphocyte ratio	1.76 (1.40–2.12)	2.36 (1.73–3.16)	2.88 (1.98–4.52)	** < 0.001**
Platelet-to-lymphocyte ratio	138 (115–155)	146 (111–187)	161 (130–199)	0.26

Data are presented as median values with interquartile ranges, unless otherwise stated. Number of pack-years was calculated as the number of cigarettes smoked per day/20 times the number of years smoked. Bold values indiactes statistical significance at p < 0.05 level.

### Patients with neovascular AMD or geographic atrophy display similar expression levels of TNF-α, IL-6, and IL-10 for different stimulation conditions when compared to a control group

Supplemental table S2 shows that LPS elicited an increase in expression of cytokines, while the effect on the increase in expression of cytokines was less consistent for CML and A2E. Table 2 shows the median expression levels of the three inflammatory cytokines, TNF-α, IL-6, IL-10, separately for the control group, the neovascular AMD and the GA group in the non-treated condition and after stimulation of inflammatory cytokines with LPS, CML, and A2E. No statistical differences were observed regarding the median expression level of TNF-α, IL-6, IL-10 between the control group, the neovascular AMD and the GA group, in the non-treated condition and after stimulation.

**Table 2:** Cytokine expression of TNF-α, IL-6, IL-10 for different stimulation conditions in patients with neovascular AMD or geographic atrophy (GA) and a control series.

	Controls (N = 30)	Neovascular AMD (N = 27)	GA (N = 24)	P value
**Non-treated**				
TNF-α	6.03 (4.01–7.23)	5.37 (3.77–7.06)	5.98 (4.84–6.97)	0.55
IL-6	4.71 (2.22–6.34)	4.23 (2.61–5.24)	4.35 (2.79–6.55)	0.34
IL-10	7.69 (6.08–8.23)	6.82 (4.85–8.86)	8.10 (6.74–9.41)	0.36
**LPS-treated**				
TNF-α	194 (72–270)	187 (112–436)	241 (162–306)	0.54
IL-6	668 (188–1212)	461 (143–870)	765 (210–1716)	0.78
IL-10	24.6 (4.77–41.8)	12.7 (8.21–36.1)	21.7 (8.50–59.0)	0.18
**CML-treated**				
TNF-α	6.39 (4.07–11.5)	6.20 (3.11–10.3)	4.95 (2.84–8.73)	0.60
IL-6	3.20 (1.28–10.7)	1.63 (0.77–4.48)	2.69 (1.26–6.72)	0.09
IL-10	5.50 (1.13–8.41)	2.16 (1.13–5.04)	3.23 (1.57–6.95)	0.10
**A2E-treated**				
TNF-α	1.02 (0.76–1.80)	1.27 (0.74–1.45)	1.19 (0.80–1.83)	0.92
IL-6	1.91 (0.75–4.42)	1.33 (0.66–2.77)	1.00 (0.67–2.58)	0.45
IL-10	1.98 (0.41–6.69)	2.74 (0.83–4.94)	1.47 (0.83–4.76)	0.89

Numbers are presented as fold-change where relative expression was calculated from the differences in cycle threshold (Ct) of the internal standard (GAPDH) compared to the target mRNA with and without treatment (ΔΔCt). Data are presented as median values with interquartile ranges. P values denote were calculated using an independent samples median test.

*AMD* age-related macular degeneration, *GA* geographic atrophy, *LPS* lipopolysaccharide, *CML* Nε-carboxymethyllysine, *A2E* *N*-retinyl-*N*-retinylidene ethanolamine, *TNF*-α tumour necrosis factor α, *IL* interleukin.

In Table 3 the results are given for the multinomial logistic regression analysis adjusted for sex and age. A higher expression of TNF-α after stimulation with A2E was observed in the GA as compared to controls, but not in the neovascular AMD group. None of the other stimulation conditions showed a significant relation between cytokine expression and neovascular AMD or GA.

**Table 3:** Fold increase of cytokine expression in patients with neovascular AMD and geographic atrophy (GA) as compared controls for different stimulation conditions.

	Neovascular AMD	P value	GA	P value
**Non-treated**				
TNF-α	0.99 (0.56–1.76)	0.98	1.28 (0.58–2.80)	0.54
IL-6	1.29 (0.72–2.32)	0.39	1.91 (0.90–4.04)	0.093
IL-10	0.64 (0.32–1.28)	0.21	1.03 (0.42–2.56)	0.95
**LPS-treated**				
TNF-α	1.30 (0.71–2.39)	0.40	1.24 (0.57–2.72)	0.59
IL-6	1.13 (0.63–2.01)	0.68	1.76 (0.75–4.13)	0.20
IL-10	0.93 (0.53–1.65)	0.81	0.74 (0.35–1.53)	0.41
**CML-treated**				
TNF-α	0.81 (0.42–1.54)	0.52	0.46 (0.20–1.06)	0.068
IL-6	0.60 (0.30–1.20)	0.15	0.77 (0.35–1.70)	0.52
IL-10	0.63 (0.34–1.17)	0.14	0.71 (0.32–1.55)	0.39
**A2E-treated**				
TNF-α	1.41 (0.63–3.14)	0.40	2.61 (1.00–6.83)	0.051
IL-6	0.82 (0.45–1.48)	0.51	0.97 (0.49–1.93)	0.93
IL-10	1.31 (0.73–2.35)	0.37	1.03 (0.49–2.19)	0.94

Data are presented as estimated odds ratios adjusted for age and sex per incremental increase in Z-score of cytokine expression with 95% confidence intervals. Reference group were healthy controls.

*AMD* age-related macular degeneration, *GA* geographic atrophy, *LPS* lipopolysaccharide, *CML* Nε-carboxymethyllysine, *A2E* *N*-retinyl-*N*-retinylidene ethanolamine, *TNF*-*α* tumour necrosis factor α, *IL* interleukin.

In Table 4 we repeated all analyses additionally adjusted for NLR. Results did not materially differ.

**Table 4:** Fold increase of cytokine expression in patients with neovascular AMD and geographic atrophy (GA) as compared controls for different stimulation conditions.

	Neovascular AMD	P value	GA	P value
**Non-treated**				
TNF-α	1.16 (0.58–2.30)	0.67	2.27 (0.82–6.23)	0.11
IL-6	1.44 (0.78–2.66)	0.24	2.30 (1.01–5.19)	**0.047**
IL-10	0.73 (0.35–1.50)	0.39	1.64 (0.50–5.34)	0.41
**LPS-treated**				
TNF-α	1.30 (0.71–2.43)	0.40	1.18 (0.53–2.66)	0.69
IL-6	1.13 (0.63–2.02)	0.69	1.71 (0.71–4.09)	0.23
IL-10	0.94 (0.53–1.67)	0.84	0.68 (0.31–1.48)	0.33
**CML-treated**				
TNF-α	0.82 (0.43–1.57)	0.56	0.41 (0.17–1.01)	0.052
IL-6	0.62 (0.31–1.23)	0.17	0.81 (0.36–1.81)	0.60
IL-10	0.66 (0.36–1.23)	0.19	0.70 (0.31–1.58)	0.39
**A2E-treated**				
TNF-α	1.45 (0.65–3.26)	0.37	2.76 (1.03–7.39)	**0.044**
IL-6	0.82 (0.45–1.48)	0.51	0.99 (0.49–1.98)	0.98
IL-10	1.39 (0.77–2.52)	0.28	0.95 (0.43–2.11)	0.90

Data are presented as estimated odds ratio adjusted for age, sex, and neutrophil-to-lymphocyte ratio per incremental increase in Z-score of cytokine expression with 95% confidence intervals. Reference group were healthy controls. Bold values indiactes statistical significance at p < 0.05 level.

*AMD* age-related macular degeneration, *GA* geographic atrophy, *LPS* lipopolysaccharide, *CML* Nε-carboxymethyllysine, *A2E* *N*-retinyl-*N*-retinylidene ethanolamine, *TNF*-α tumour necrosis factor α, *IL* interleukin.

## Discussion

The purpose of this study was to test the hypothesis that A2E contributes to the elevated inflammatory activity in AMD. Therefore, we measured the expression of cytokines TNF-α, IL-6, IL-10 in whole blood samples after stimulation with A2E in a population of patients with neovascular AMD and GA in comparison to persons without AMD. As it has been convincingly demonstrated that monocytes mount an inflammatory in response to PAMPs^36^, we hypothesized that monocytes would be the primary cell type responsible for the inflammatory response to A2E exposure. This reasoning lines up with our choice of whole blood ex vivo stimulation, as this was shown to be a reasonable proxy for monocytic cytokine secretion^37^. Admittedly, some evidence exists, that neutrophils can express both anti- and pro-inflammatory cytokines in response to PAMPs as well^38^. The methodology used in our study does not allow us to differentiate between their respective contribution. Cytokine detection by soluble protein measurements, such as ELISA is the most widely used method, but can be hampered by its detection range, which remains an obstacle in stimulation assays^39–42^. The RT-qPCR-based whole blood ex vivo immune stimulation assay was based on the similar approach using ELISA and LPS for detection^6,33^. We aimed to produce a whole blood ex vivo immune stimulation assay for a larger range of detection, without the need for purification or dilution. RNA expression assays are indeed more sensitive, but with an increased risk of type 1 errors. Our results did not show statistical differences in the median cytokine expression levels in the neovascular AMD and the GA group as compared to the control group after stimulation with A2E. Likewise, the median cytokine expression levels were not significantly different in the neovascular AMD and the GA group when compared to the control group after stimulation with CRP and CML. A significant association between higher TNF-α expression after A2E stimulation was found for the GA group. However, the 95% confidence interval was broad, indicating that this estimate was imprecisely measured. Previously, others have shown that A2E treatment of RPE cells induces increased expression of a range of proinflammatory cytokines^25,43^. This study is, to our knowledge, the first to use an ex vivo A2E stimulation assay in whole blood. The A2E does not give an inflammatory response in our cytokine panel after 4 h of stimulation. Others have incubated cells for up to 4 days, which was not possible in our setup with ex vivo non dividing blood cells. One group treated ARPE cells with 25 µM A2E for 24 h and saw increased protein levels of IL-6, TNF-α and other interleukins^43^. Another group treated leukocyte-derived IPSC-RPE with 10 µM A2E for 4 days. Samples were analysed with RT-qPCR, and they found an increased pro-inflammatory response, including IL-6^25^. A few drawbacks should be considered. Although RPE is the main pathogenic target of macular degeneration, it is challenging to obtain an adequate number of RPE cells from AMD patients for disease modelling. The ARPE-19 cell line was obtained from a 19-year-old donor and are the most widely used cells for studying in vitro AMD. However, whether ARPE-19 cells epitomize the characteristics of human RPE cells or not is debatable^25^. We investigated the possibility of our study being underpowered and therefore unable to detect true difference between the groups. However, a post-hoc power analysis using an Altman nomogram found that this study should be able to minimally detect a standardized difference of 0.6 in GA and 0.8 in neovascular AMD with a 20% chance of a type II error.

Therefore, we conclude that if A2E indeed substantially contributes to the inflammatory component of AMD, our sample size should be enough to pick up a consistent trend, which is not apparent from our data. Contrastingly, our sample size was sufficient to detect a significant difference in terms of NLR between the groups.

With due caution, the results from our study do not support the hypothesis, that A2E contributes to heightened inflammatory activity in AMD.

## Material and methods

### Design

This study was conducted as a prospective case–control study. The study is approved by the Regional Committee of Ethics in Research of the Region of Zealand (SJ-385, SJ-510). Informed consent was obtained from all participants prior to inclusion. The project adhered to the tenets of the Declaration of Helsinki.

### Inclusion and participant eligibility

A population of 60 patients with late-stage AMD (30 patients with neovascular AMD and 30 patients with GA secondary to AMD) were included from the outpatient program at Department of Ophthalmology, Zealand University Hospital (ZUH). Patients’ spouses accompanying them in the clinic were included as healthy controls. This strategy served to include controls that matched patients concerning age and environmental exposures. Individuals were not included if they had any infections, cancer, inflammatory or autoimmune diseases, or if they received immune-modulating medication for any reason. Patients with neovascular AMD all received treatment with intravitreal anti-vascular endothelial growth factor but were not included within 4 or 8 weeks from last treatment with ranibizumab or aflibercept, respectively. This restriction served to avoid potential interaction of systemic uptake of antibodies. Detailed diagnosis of patients with neovascular AMD with angiography was performed at referral in treatment-naïve eyes. We did not recruit newly diagnosed patients at their initial visit, since we have previously found that recent onset of choroidal neovascularization is associated with acute immune activity^44^.

### Retinal diagnosis

All participants underwent comprehensive ocular examination. They had performed ophthalmoscopic fundus examination, spectral-domain optical coherence tomography and autofluorescence imaging (Spectralis HRA-OCT, Heidelberg Engineering, Heidelberg, Germany). All patients with neovascular AMD had performed fluorescein and indocyanine green angiography to confirm the diagnosis. Best corrected visual acuity was measured according to the Early Treatment Diabetic Retinopathy Study^45^. Participant’s retinal images were graded, and participants were categorized in the following categories:

- Healthy controls had no drusen or < 10 small drusen without any pigment abnormalities in either eye.
- Patients with GA had drusen maculopathy and areas of GA with involvement of the macula centre in one or both eyes. Patients with current or former choroidal neovascularization, or any sign of this (i.e. fibrosis, fluid, haemorrhages) in any eye, was not included to this study group.
- Patients with neovascular AMD had fibrovascular RPE detachments and active choroidal neovascular membranes with subretinal haemorrhages, fluid or fibrosis.

### Biosynthesis of *N*-retinylidene-*N*-retinylethanolamine (A2E).

A2E was synthesized in a reaction biomimicking the visual cycle as described by Park et al*.* with minor revisions^46^ and measured by high performance liquid chromatography (HLPC). The analyses were monitored with an UV–visible detector set at 275 nm, with a universal column (Tricorn 5/50 Column, GE Healthcare Chicago, USA), and a flow rate of 0.8 ml/min. As the classical purification of A2E by HPLC is difficult due to its amphiphilic nature, we employed a cation exchange resin for the separation of A2E from the crude reaction mixture as previously described^47,48^. In brief, two equivalents all-*trans*-retinal (ATR) and one equivalent ethanolamine were mixed in ethanol with acetic acid and left to stir for 3 days, and hereafter desiccated on a centrifugal vacuum concentrator (miVac, Thermo Fisher Scientific, Waltham, USA), and re-dissolved in of acetonitrile (80%, Sigma-Aldrich). The crude reaction mixture was loaded on a weak acid resin (Amberlite, Sigma-Aldrich, St. Louis, USA) and eluted with 80% HLPC-grade methanol (Sigma-Aldrich, St. Louis, USA) with sodium hydroxide (pH 12), followed by 100% methanol, and then 100% methanol with 0.1% trifluoroacetic acid (TFA) in sequence. A2E (and iso-A2E) were eluted only with the 100% methanol solution containing TFA^46^. The A2E residue was diluted to 40 mM in DMSO (Sigma-Aldrich, St. Louis, USA) and stored in the dark at 4 °C. Fresh A2E solutions were prepared from the A2E stock and diluted to a final concentration of 20 μM in preheated Roswell Park Memorial Institute (RPMI)-1640 media (Life technologies, Thermo Fisher Scientific, Waltham, USA) Manipulations involving A2E addition, were conducted under dimmed light.

### Blood collection and stimulation

Blood was sampled from the antecubital vein in two tubes: one 10 mL lithium-heparin-coated vacutainer for cell stimulation, and one 5,5 mL EDTA-coated vacutainer was analysed for complete blood cell count, red blood cells, leukocytes, and platelets using a Sysmex XN automated haematology analyser XN (Sysmex Corporation, Kobe, Japan). In brief, blood-samples from lithium-heparin tubes were diluted 1:1 in RPMI-1640 containing LPS, CML, A2E or DMSO as a mock control and incubated in a humidified 37 °C, 5% CO_2_ incubator for 4 h. LPS (from *E.* *coli* serotype O26:B6, Sigma-Aldrich, St. Louis, USA) was diluted to 5 mg/ml in DMSO and added to the blood-RPMI solution to obtain 100 ng/ml concentrations. CML (Carboxy-methyl Lysine modified Bovine Serum Albumin, PAB-26388-500, Nordic Biosite, Täby, Sweden) in DMSO and added to blood samples to a final concentration of 10 µg CML/ml, and A2E was added to blood cells as described above.

### RNA purification and determination of cytokine expression levels by quantitative real-time PCR

Blood fractioning was performed in order to separate plasma from cells. Stimulated blood samples were centrifuged at 400×*g* for 5 min and the supernatant was transferred to a new tube and spun at 4600×*g* for 10 min. Total RNA was extracted from 1 ml of the bottom fraction with the GeneJET RNA Purification Kit (Life Sciences, Thermo FishEr, Waltham, USA), quantified on a microplate spectrophotometer (Epoch, Biotek, Winooski, United States), and 50 ng was reverse transcribed using the FIREScript RT cDNA synthesis kit (Solis BioDyne, Tartu, Estonia). mRNA levels was analysed by RT-qPCR with a StepOnePlus real-time PCR system (Applied Biosystems, Foster City, USA) as described previously^49^, using HOT FIREPol EvaGreen qPCR Mix (Solis BioDyne, Tartu, Estonia) with a hot start at 95 °C followed by 40 cycles of 95 °C for 20 s, 58 °C for 40 s, and 72 °C for 20 s, followed by a melting curve analysis, using SYBR green fluorescence reading as described elsewhere^50^. Differences between patient groups and treatments were determined by threshold cycles (Ct) values as described in detail elsewhere^49^. In brief, data is presented as fold change of the target gene expression. First, the difference between treated and untreated target gene (treated–untreated target samples = Δ target) and between treated and untreated reference gene (treated–untreated reference samples = Δ control) are calculated. Next, the difference between Δ target and Δ control are calculated to arrive at the ΔΔCt value. The ΔΔCt value are given in minus logarithm base 2 to get expression in fold change^49^. All assays were performed in technical triplicate on a MicroAmp Fast Optical 96-Well Reaction Plate with MicroAmp Optical Adhesive Film (Life Sciences, Thermo Fisher, Waltham, USA). Relative expression of genes was normalized to the housekeeping gene GAPDH. The Primers were designed with Primer3 and BLAST (ncbi.nlm.nih.gov/tools/primer-blast) and are listed in Table 5. All analyses were performed in one batch, precluding any “batch effects”.

**Table 5:** Primers for RT-qPCR.

Target mRNA	NM	Forward primer	Reverse primer	Amplicon (bp)
GAPDH	NM_001357943.2	ATGGGGAAGGTGAAGGTCG	GGGGTCATTGATGGCAACAATA	66
TNF-α	NM_000594.3	CTCACATACTGACCCACGGC	ACGTCCCGGATCATGCTTTC	78
IL-6	NM_000600.5	TTCGGTCCAGTTGCCTTCTC	TCTTCTCCTGGGGGTACTGG	80
IL-10	NM_000572.2	AGCCTGACCACGCTTTCTAG	GGGCTCCCTGGTTTCTCTTC	123

Primers for each gene of interest were designed for each target mRNA (TNF-α, IL-6, IL-10) and the internal standard (GAPDH).

### Outcomes

The expression levels of the three inflammatory cytokines, TNF-α, IL-6, IL-10, separately for the control group, the neovascular AMD and the GA group in the non-treated condition and after stimulation of inflammatory cytokines with LPS, CML, and A2E were the primary outcomes in this study.

### Statistical analyses

The number of observations included in the statistical analyses are shown in supplementary Table S1. If the handling time exceeded 5 h from venepuncture, the samples were excluded from downstream analysis. Ct values with a difference above 0.5 between technical replicates were omitted from the study. Likewise, if the RNA was degraded (based on spectrophotometry), samples were discarded. Differences in continuous outcomes between the three categories (controls, geographic atrophy, and neovascular AMD) were tested using an independent samples Kruskal–Wallis test. Differences in dichotomous variables were tested using a Pearson Chi square test. If the overall difference was found to be statistically significant (P value < 0.05), two independent samples Mann–Whitney U tests were performed to identify where the significant difference was. Analyses of inflammatory cytokines expression before and after stimulation were analysed using a dependent samples Wilcoxon signed rank test. We also performed a multinomial logistic regression analysis with controls, neovascular AMD and GA as dependent dichotomous and standardized expression values of the three cytokines under non treated conditions, after stimulation with LPS, CML, and A2E as independent variables. All odds ratios were calculated with the control group as the reference category. Analyses were adjusted for sex and age and additionally for sex, age and NLR. As this is an exploratory rather than confirmatory study, we refrain from correction for multiple testing.

## Supplementary Information

Supplementary Information 1.

Supplementary Information 2.
