# Supplementary material for: EX-vivo whole blood stimulation with A2E does not elicit an inflammatory cytokine response in patients with age-related macular degeneration
Source: Sci Rep. 2021 Apr 15;11:8226. doi: 10.1038/s41598-021-87337-1 (PMC8050255; doi:10.1038/s41598-021-87337-1)
Supplement: Supplementary file 1 — Supplementary Information 1. [file 41598_2021_87337_MOESM1_ESM.docx]

**Table S1. Fold increase of cytokine expression in patients with neovascular AMD and geographic atrophy (GA) as compared controls for different stimulation conditions.**

|  | **Neovascular AMD** | **P value** | **GA** | **P value** |
| --- | --- | --- | --- | --- |
| ***Non-treated*** |  |  |  |  |
| TNF-α | 1.50 (0.61 – 3.68) | 0.37 | 2.83 (0.85 – 9.44) | 0.09 |
| IL-6 | 1.55 (0.72 – 3.36) | 0.27 | 2.40 (0.92 – 6.26) | 0.07 |
| IL-10 | 0.54 (0.19 – 1.52) | 0.25 | 1.05 (0.26 – 4.14) | 0.95 |
|  |  |  |  |  |
| ***LPS-treated*** |  |  |  |  |
| TNF-α | 1.08 (0.50 – 2.35) | 0.85 | 0.97 (0.35 – 2.69) | 0.96 |
| IL-6 | 0.91 (0.46 – 1.78) | 0.77 | 1.60 (0.57 – 4.46) | 0.37 |
| IL-10 | 0.77 (0.38 – 1.54) | 0.46 | 0.56 (0.22 – 1.41) | 0.22 |
|  |  |  |  |  |
| ***CML-treated*** |  |  |  |  |
| TNF-α | 0.87 (0.41 – 1.89) | 0.73 | 0.36 (0.12 – 1.06) | 0.064 |
| IL-6 | 0.56 (0.22 – 1.43) | 0.22 | 0.83 (0.29 – 2.40) | 0.73 |
| IL-10 | 0.55 (0.25 – 1.21) | 0.14 | 0.59 (0.22 – 1.59) | 0.30 |
|  |  |  |  |  |
| ***A2E-treated*** |  |  |  |  |
| TNF-α | 1.04 (0.40 – 2.72) | 0.94 | 1.95 (0.62 – 6.13) | 0.25 |
| IL-6 | 0.47 (0.20 – 1.09) | 0.078 | 0.50 (0.19 – 1.30) | 0.15 |
| IL-10 | 1.15 (0.58 – 2.31) | 0.69 | 0.71 (0.28 – 1.80) | 0.48 |

Data are presented as estimated odds ratio adjusted for age, sex, smoking status (current, previous, never) and neutrophil-to-lymphocyte ratio per incremental increase in Z-score of cytokine expression with 95% confidence intervals. Reference group were healthy controls. AMD: Age-related macular degeneration; GA: geographic atrophy; LPS: lipopolysaccharide; CML: Nε -carboxymethyllysine; A2E: N-retinyl-N-retinylidene ethanolamine; TNF- α: tumour necrosis factor α; IL: interleukin.

| IL-10 | 0.15 (-0.04 – 0.17) | 0.20 |
| --- | --- | --- |
