# Supplementary material for: EX-vivo whole blood stimulation with A2E does not elicit an inflammatory cytokine response in patients with age-related macular degeneration
Source: Sci Rep. 2021 Apr 15;11:8226. doi: 10.1038/s41598-021-87337-1 (PMC8050255; doi:10.1038/s41598-021-87337-1)
Supplement: Supplementary file 2 — Supplementary Information 2. [file 41598_2021_87337_MOESM2_ESM.docx]

**Table S2. Association between smoking pack years and fold increase of cytokine expression in whole study sample combined for different stimulation conditions. Data are presented as change in fold increase of the cytokines per smoked pack year with 95% confidence intervals.**

| ***Pack years*** | **All groups** | **P value** |
| --- | --- | --- |
| ***Non-treated*** |  |  |
| TNF-α | 0.07 (-0.19 – 0.05) | 0.39 |
| IL-6 | 0.10 (-0.19 – 0.05) | 0.93 |
| IL-10 | -0.03 (-0.04 – 0.03) | 0.77 |
|  |  |  |
| ***LPS-treated*** |  |  |
| TNF-α | 0.06 (-3.12 – 5.58) | 0.58 |
| IL-6 | -0.12 (-36.27 – 10.73) | 0.28 |
| IL-10 | -0.12 (-2.74 – 0.83) | 0.29 |
|  |  |  |
| ***CML-treated*** |  |  |
| TNF-α | 0.13 (-0.09 – 0.32) | 0.27 |
| IL-6 | -0.11 (-0.34 – 0-12) | 0.34 |
| IL-10 | -0.04 (-0.14 – 0.10) | 0.73 |
|  |  |  |
| ***A2E-treated*** |  |  |
| TNF-α | 0.23 (0.01 – 0.52) | ***0.046*** |
| IL-6 | 0.24 (0.04 – 1.37) | ***0.038*** |
